# Anti-CD47 Monoclonal Antibody–Drug Conjugate: A Targeted Therapy to Treat Triple-Negative Breast Cancers

**DOI:** 10.3390/vaccines9080882

**Published:** 2021-08-10

**Authors:** Yingnan Si, Ya Zhang, Jia-Shiung Guan, Hanh Giai Ngo, Angela Totoro, Ajeet Pal Singh, Kai Chen, Yuanxin Xu, Eddy S. Yang, Lufang Zhou, Runhua Liu, Xiaoguang (Margaret) Liu

**Affiliations:** 1 Department of Biomedical Engineering, University of Alabama at Birmingham (UAB), 1825 University Blvd, Birmingham, AL 35294, USA; yingnan@uab.edu (Y.S.); yazhang9@uab.edu (Y.Z.); guan0926@uab.edu (J.-S.G.); hanh96@uab.edu (H.G.N.); angmshel@uab.edu (A.T.); ajeeetts@uab.edu (A.P.S.); kaisdzb@uab.edu (K.C.); lfzhou@uab.edu (L.Z.); 2 Department of Medicine, University of Alabama at Birmingham (UAB), 703 19th Street South, Birmingham, AL 35294, USA; yuanxin8@uab.edu; 3 Department of Radiation Oncology, University of Alabama at Birmingham (UAB), 1808 7th Avenue South, Birmingham, AL 35294, USA; shyang@uabmc.edu; 4 O’Neal Comprehensive Cancer Center, University of Alabama at Birmingham (UAB), 1824 6th Avenue South, Birmingham, AL 35233, USA; 5 Department of Genetics, University of Alabama at Birmingham (UAB), 702 20th St., Birmingham, AL 35233, USA; runhua@uab.edu

**Keywords:** triple-negative breast cancers, targeted therapy, CD47 receptor, monoclonal antibody, antibody–drug conjugate

## Abstract

Triple-negative breast cancers (TNBCs) are frequently recurrent due to the development of drug resistance post chemotherapy. Both the existing literature and our study found that surface receptor CD47 (cluster of differentiation 47) was upregulated in chemotherapy-treated TNBC cells. The goal of this study was to develop a monoclonal antibody (mAb)-based targeting strategy to treat TNBC after standard treatment. Specifically, a new mAb that targets the extracellular domain of receptor CD47 was developed using hybridoma technology and produced in fed-batch culture. Flow cytometry, confocal microscopy, and in vivo imaging system (IVIS) showed that the anti-CD47 mAb effectively targeted human and mouse TNBC cells and xenograft models with high specificity. The antibody–drug conjugate (ADC) carrying mertansine was constructed and demonstrated higher potency with reduced IC_50_ in TNBC cells than did the free drug and significantly inhibited tumor growth post gemcitabine treatment in MDA-MB-231 xenograft NSG model. Finally, whole blood analysis indicated that the anti-CD47 mAb had no general immune toxicity, flow cytometry analysis of lymph nodes revealed an increase of CD69^+^ NK, CD11c^+^ DC, and CD4^+^ T cells, and IHC staining showed tumoral infiltration of macrophage in the 4T1 xenograft BALB/cJ model. This study demonstrated that targeting CD47 with ADC has great potential to treat TNBCs as a targeted therapy.

## 1. Introduction

The highly aggressive heterogeneous triple-negative breast cancers (TNBCs, HER2^−^/ER^−^/PR^−^) account for 15–20% of breast cancers. The standard cytotoxic chemotherapies, e.g., anthracycline-taxane and gemcitabine (GC)-paclitaxel [1,2,3,4], are currently the main systemic treatment options. However, TNBCs usually develop drug resistance and result in distant metastasis post primary treatment and chemotherapy, which could be caused by ATP-binding cassette transporters-mediated drug efflux [5,6,7], cancer stem cells [8,9], hypoxia [10,11], dysregulation of apoptosis [12,13], heterogeneity [14,15], activation of survival, growth and invasion signaling pathways [15], or others [3,4]. Moreover, the adverse effects, early relapse, high recurrence rate (>50%), and poor survival [2,3,4,7] have significantly reduced the clinical benefits of chemotherapies [16,17]. Thus, a targeted treatment strategy is needed for TNBC especially after standard therapies.

**Figure 1:**
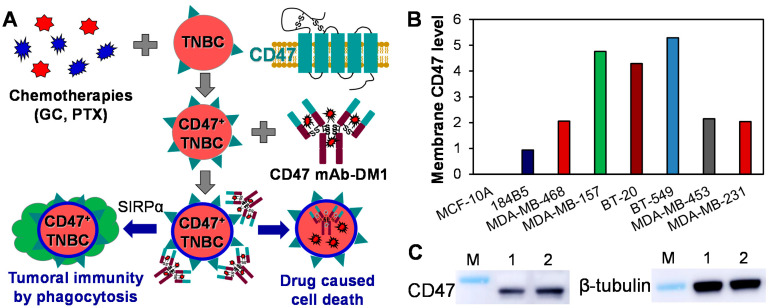
Surface receptor CD47 in TNBC. (**A**) Anti-TNBC mechanisms by targeting CD47-overexpressing TNBC cells to deliver payload with ADC and reactivating macrophage to induce phagocytosis-based TNBC cell death. (**B**) Relative expression of membrane CD47 receptor in normal breast cells (MCF-10A and 184B5) and TNBC cells (MDA-MB-468, MDA-MB-157, BT-20, BT-549, MDA-MB-453, and MDA-MB-231). The CD47 expression was analyzed using MS analysis and presented as peptide counts. (**C**) Western blotting analysis of CD47 in MDA-MB-231 cells (Lane: 1) and GC treated MDA-MB-231 cells (Lane: 2).

The CD47 receptor is highly expressed on tumor cells and provides a “don’t eat me” signal via interacting with the N-terminus of signal regulatory protein alpha (SIRPα) on macrophages and other myeloid cells [18,19] to block phagocytosis [20,21]. Disrupting the CD47-SIRPα ligand promotes the phagocytosis of tumor cells [22] and immunity-mediated antitumor effects [23,24,25,26,27,28] (Figure 1A). Furthermore, the literature has reported that the surface expression of glycoprotein CD47 in TNBC can be upregulated by chemotherapies (carboplatin, doxorubicin, GC, and paclitaxel) [29,30], but it is not detectable or low in important organs or normal breast tissues [27,31]. We also observed that GC-resistant TNBC cells had higher CD47 expression than did drug-sensitive TNBCs. All these studies indicated that CD47 is an ideal target to treat drug-resistant or chemotherapy-treated TNBCs.

Targeted monoclonal antibodies (mAbs) and antibody–drug conjugates (ADCs) have been developed to treat various solid tumors [32,33,34,35,36,37,38,39]. The mAbs targeting epidermal growth factor receptor (EGFR) [40,41] and folate receptor [42,43] have been developed and evaluated for TNBC treatment, but their anti-tumor efficacy is poor in clinical trials. Recently, the FDA approved the combination of Atezolizumab (immunotherapy) and Abraxane (chemotherapy) as a therapy to treat PD-L1^+^ TNBC [44,45,46,47] and Sacituzumab govitecan (ADC) to treat trophoblast cell-surface antigen 2 (Trop-2)^+^ TNBC [48,49,50,51,52]. Combining the cancer specificity of antibody treatment and high cytotoxicity of chemotherapy, ADCs have been investigated by us and others [53,54]. Despite this progress, drug resistance and high recurrence in TNBC patients remain challenging.

The objective of this study was to develop an innovative targeted ADC to treat TNBCs post chemotherapy. An anti-CD47 mAb (IgG2b/kappa) that targets the extracellular domain (Gln19-Glu141) of the cell surface receptor CD47 was developed. The ADC was constructed by conjugating our mAb with the FDA approved cytotoxic payload, mertansine (DM1), which blocks microtubulin polymerization and inhibits TNBC cell growth. Our preliminary data showed that DM1 has high cytotoxicity to TNBC cells. The specific targeting, drug delivery, and anti-tumor efficacy of the anti-CD47 ADC were investigated in vitro and in vivo. Furthermore, immune response analysis detected an increase of immune cells in lymph nodes and infiltration of macrophage in tumor but no general immune toxicity in the immunocompetent xenograft model. The developed anti-CD47 ADC could provide a novel, promising, and targeted treatment, with further development, to potentially treat drug-resistant TNBCs.

## 2. Materials and Methods

### 2.1. Cell Lines and Media

Multiple human TNBC cell lines including MDA-MB-468, MDA-MB-231 (ATCC, Manassas, VA, USA), and MDA-MB-231-FLuc (GenTarget, San Diego, CA, USA), mouse TNBC cell line 4T1-Luc (ATCC), and normal breast epithelium cell lines 184B5 and MCF-10A were used in the in vitro or in vivo evaluation studies of the developed anti-CD47 mAb and ADC. The TNBC cells were maintained in DMEM/F12 medium supplemented with 4 g/L of glucose, 4 mM of L-glutamine, and 10% fetal bovine serum (FBS, *v*/*v*) in T25, T75, or T175 flasks. The normal 184B5 and MCF-10A cells were maintained in MEGM bullet kit growth medium (Lonza, Walkersville, MD, USA) supplemented with 5% FBS. The hybridoma cells producing anti-CD47 mAb were cultivated in DMEM with 4 g/L of glucose, 4 mM of L-glutamine, and 10% FBS in T flasks, or adapted in Hybridoma-SFM (serum free medium) in shaker flasks with an agitation of 130 rpm. The seed cultures were incubated at 37 °C and 5% CO_2_ in a humidified incubator (Caron, Marietta, OH, USA). All media, supplements and bioreagents used in this study were purchased from Fisher Scientific (Waltham, MA, USA) unless otherwise specified. 

### 2.2. Anti-CD47 mAb Development and Production

The mAb that targets the first extracellular domain (Gln19-Glu141) of membrane CD47 was developed using hybridoma technology as we published previously [39]. Briefly, mFc-fused peptide (Gln19-Glu141) was expressed in HEK293 cells through transient transfection, purified using protein G, and used to immunize mice. After detecting anti-CD47 mAb in serum, the splenocytes were harvested and fused with myeloma Sp2/0-Ag14 to generate hybridoma. The top clone was screened using ELISA and adapted to serum-free suspension culture to produce mAb [55]. The anti-CD47 mAb was produced in 30–1000 mL culture at a temperature of 37 °C and agitation at 80–130 rpm. The bioproduction was seeded with a viable cell density (VCD) of 0.3–0.5 × 10^6^ cells/mL in Hybridoma-SFM fed with glucose and L-glutamine to maintain the culture concentration within 2–4 g/L and 2–4 mM, respectively. The produced mAb was purified using liquid chromatography system (Bio-Rad, Hercules, CA, USA) equipped with a Protein A column (i.e., Bio-Scale Mini UNOsphere SUPrA affinity chromatography cartridges, Bio-Rad) [56], and characterized using SDS-PAGE. The isotype of the CD47 mAb was determined using a mouse antibody isotyping kit (Sigma, St. Louis, MO, USA).

### 2.3. ADC Construction and Analysis

The anti-CD47 mAb-DM1 was constructed following our published conjugation procedure [39,54,57] with optimized conditions. Briefly, 10 mg/mL CD47 mAb, 22.9 mM of Sulfo-SMCC linker, and 10 mM of DM1 stocks were mixed with a molar ratio of 1:14:18.2. The working concentration of antibody was 1 mg/mL. After a 2-hr reaction at room temperature, the free linkers and unconjugated drug were removed with 10 kDa MWCO PES concentrator, and the unconjugated mAb and constructed ADC were further purified by a liquid chromatography system with protein A column. The system was equilibrated with buffer A composed of 0.02 M sodium phosphate and 0.02 M sodium citrate at pH 7.5, and the anti-CD47 mAb/ADC were eluted with buffer B containing 0.02 M sodium citrate and 0.1 M sodium chloride at pH 3.0. The purified ADC was neutralized to pH 7.0 with 1 M Tris solution, desalted, buffer exchanged using 10 kDa MWCO PES concentrator, sterilized, and mixed with 0.02% sodium azide for long-term storage at −80 °C. The purity and drug–antibody ratio (DAR) analysis was performed using HPLC (Shimadzu, Columbia, MD, USA) equipped with a MAbPac HIC-Butyl column (5 µm, 4.6 × 100 mm). The mobile phase A, composed of 2 M ammonium sulfate and 100 mM sodium phosphate at pH 7.0, and B, composed of 100 mM sodium phosphate at pH 7.0 were applied at room temperature with a flow rate of 1.0 mL/min. The same HPLC column and mobile phases can be used to isolate ADC from unconjugated mAb if the antibody–drug conjugation efficiency is <95%. The integrity of ADC was confirmed with SDS-PAGE.

### 2.4. SDS-PAGE and Western Blots

The non-reducing SDS-PAGE was run by loading 2 µg of mAb or ADC to Bolt™ 4 to 12% Bis-Tris Mini Protein Gel (1.0 mm) to validate the purity and integrality of the produced anti-CD47 mAb and ADC. In Western blotting analysis, 30 µg of total cell lysis protein was loaded onto the gel and electro-transferred to the PVDF membrane using PowerEase^TM^ Touch 350W Power Supply (Fisher). After blocking, the blotted membrane was probed using primary rabbit anti-mouse antibody and HRP-conjugated secondary anti-rabbit antibody (Abcam, Cambridge, UK). The blotted membrane was treated with Luminata Forte Western HRP substrate (Millipore, Boston, MA, USA). The SDS-PAGE and Western blots were imaged with a MyECL imager and quantified using ImageJ software.

### 2.5. Flow Cytometry

The flow cytometry was conducted to analyze the anti-CD47 mAb’s binding rate to TNBC cells and quantify the immune cells using a BD LSRII flow cytometer (BD Biosciences, San Jose, CA, USA). In surface binding analysis, the produced CD47 mAb was labelled with an Alexa Fluor™ 647 labelling kit (Life Technologies, part of Fisher) and used to stain MDA-MB-468, MDA-MB-231, 4T1, and 184B5 cells with 5 µg of mAb-AF647 per million of cells at room temperature for 30 min [57,58,59]. The harvested lymph nodes were dissociated with a Tissue Dissociation/Single Cell Isolation Kit (101 Bio LLC, Sunnyvale, CA, USA) following the manufacturer’s procedure, and stained with Cy5 anti-CD69 antibody, APC anti-CD11c antibody, or PE/Cy7 anti-CD4 antibody (BioLegend, San Diego, CA, USA) for flow cytometry analysis.

### 2.6. Live-Cell Confocal Imaging

Confocal microscopy imaging was collected to validate the targeting and internalization of anti-CD47 mAb in TNBC cells following our reported protocol [39,54,58,59]. Briefly, the MDA-MB-468 cells were stained with BacMam GFP Transduction Control, which expresses eGFP protein in the cytoplasm, NucBlue Live ReadyProbes to stain the nucleus, and AF647 labelled anti-CD47 mAb to target TNBC cells via surface receptors. The live-cell images were collected using a Nikon A1R-HD25 confocal microscope with a high-speed resonance scanner (Nikon USA, Melville, NY, USA).

### 2.7. CD47 Binding Affinity Analysis

The binding affinity of the mAb-CD47 receptor was tested by measuring the dissociation constant (K_D_) following the reported procedure [60]. Briefly, the 96-well plate was coated with 200 ng of CD47 receptor in 100 µL of coating buffer (50 mM carbonate-bicarbonate buffer, pH 9.6) and incubated at 4 °C overnight. The plate was washed three times with PBS containing 0.05% Tween 20 and blocked with 2% BSA (bovine serum albumin). Then, 100 µL of three equilibrated reactions of anti-CD47 mAb and receptor with nanomolar ratios of 2.22:4.44, 3.33:3.33, and 4.44:2.22 and anti-CD47 mAb with concentrations of 0, 1, 5, 10, 20, 40, 80, 160, 320, and 640 nM (for standard curve generation) were added into each well. After incubating at 37 °C for 1 h, 100 µL of HRP goat anti-mouse IgG secondary antibody (Abcam, Cambridge, MA, USA) at a concentration of 50 ng/mL was added to each well and incubated at 37 °C for 1 h and followed with TMB substrate for color development. The reaction was stopped by adding 100 µL of 1 M sulfuric acid. The absorbance was recorded with a BioTek plate reader at a wavelength of 450 nm.

### 2.8. In Vitro Cytotoxicity Assay

The 96-well plates were seeded with MDA-MB-231 and MDA-MB-468 cells at a VCD of 5 × 10^5^ cells/mL in 100 µL of DMEM/F12 complete medium with triplication. Then, 100 μL of cell growth medium containing ADC or free drug was added to reach final concentrations of 0–200 nM and the cells were incubated in a CO_2_ incubator for 72 h. The cytotoxicity of ADC or free drug was measured with a CellTiter-Glo Luminescent Cell Viability Assay (Promega, Madison, MI, USA) and reported as the relative viability using untreated cells as the control. The IC_50_ value was calculated using the ED50V10 Excel add-in.

### 2.9. Whole Blood Analysis

The blood samples were extracted by cardiac puncture for whole blood analysis using HemaVet 950FS (Drew Scientific, Miami Lakes, FL, USA). The leukocytes (white blood cell, neutrophil, lymphocyte, monocyte, eosinophils), erythrocytes (red blood cell, hemoglobin, mean corpuscular hemoglobin), and thrombocytes (platelet) were titrated to analyze the general peripheral immune response.

### 2.10. Primary TNBC Xenograft Model Generation

A human TNBC xenograft mouse model was generated by subcutaneously (s.c.) injecting 3 × 10^6^ of MDA-MB-231-FLuc cells into 6-week-old NSG (NOD scid gamma) female mice (Jackson Labs, Bar Harbor, ME, USA). This immunocompromised model was used to evaluate the tumor targeting of our mAb and anti-TNBC efficacy of the constructed ADC. The immunocompetent mouse model was generated by injecting 1 × 10^6^ of mouse TNBC 4T1-FLuc cells into the BALB/cJ female mice, which was used to analyze the tumoral immunity. Mice developed tumors within 2 weeks post cells injection.

### 2.11. In Vivo Imaging System (IVIS) Imaging

When TNBC tumor volume reached ~100 mm^3^, 50 µg of Cyanine 5.5 (Cy5.5, Lumiprobe, Hunt Valley, MD, USA) labelled anti-CD47 mAb or 1.5 nmole Cy5.5 (control, similar amount as labelled Cy5.5 in mAb) was intravenously (i.v.) administrated via tail vein for TNBC-targeting analysis. Mice were imaged at 24 h post mAb injection under IVIS Lumina Series III (PerkinElmer, Waltham, MA, USA) with a wavelength of 660 nm/710 nm (excitation/emission) and exposure time of 10 s. Inhalation anesthesia was induced by delivering 3.5–4.5% isoflurane in oxygen to mice via the respiratory system (nose cone), then maintained with a concentration of 1–2% during imaging procedure.

### 2.12. In Vivo Anti-TNBC Efficacy Study

The TNBC MDA-MB-231-FLuc xenograft mice were treated with 16 mg/kg BW of GC by intraperitoneal (IP) injection when tumor volume reached ~50 mm^3^. When tumor volume reached ~100 mm^3^, mice were randomized into four groups (*n* = 5) and treated with i.v. administration of PBS, 8 mg/kg of CD47 mAb, and 8 mg/kg and 24 mg/kg of anti-CD47 ADC on a Q4Dx4 schedule (4-day interval for 4 injections). Tumor volume was measured using an electronic caliper and calculated as “width × width × length/2” in mm. Body weight was measured every other day. The in vivo treatment study was ended after 21 days of treatment, which was determined based on our previous animal study of ADC treatment [54,56,57]. At the end of the experiment, mice were euthanized and the TNBC tumors were collected to measure the wet weight.

### 2.13. Immunohistochemistry (IHC) Staining and Hematoxylin and Eosin (H&E) Staining

The IHC and H&E staining were performed as described in our previous publication [61]. In IHC staining, the paraffin-embedded slides were immersed in xylene followed by 3% H_2_O_2_ in PBS, and they were blocked with 3% normal goat serum. The rabbit anti-mouse CD11b mAb (Cell Signaling, Danvers, MA, USA) and HRP-conjugated goat anti-rabbit IgG mAb were used to stain the macrophages in the tumor tissue. After color development with DAB chromogen, the slides were counterstained with hematoxylin and dehydrated in absolute ethanol. In H&E staining, the embedded normal organ slides were dewaxed, hydrated with 100-0% ETOH, immersed in hematoxylin solution, and rinsed with tap water and eosin. After equilibration in ethanol, the stained slides were dipped in xylene and mounted with cytoseal Xyl.

### 2.14. Statistical Analysis

The in vivo sample size was determined following our previous ADC therapy studies [39,54]. All numerical data were presented as mean ± standard error of the mean (SEM). The significance of differences among groups was analyzed using a one-way ANOVA followed by post-hoc (Dunnett’s) analysis. Statistical analysis was performed using GraphPad Prism and * *p* < 0.005 was considered for all tests.

## 3. Results and Discussion

Both the existing literature [29,30] and our study revealed that the expression of surface receptor CD47 is upregulated post chemotherapy. This study developed and evaluated an anti-CD47 mAb-based ADC to treat TNBCs via surface targeting of CD47 to deliver potent DM1 intracellularly. The immune response post ADC injection was also observed.

### 3.1. CD47 Expression in TNBC

The surface expression of CD47 in TNBC cell lines was analyzed by titrating the peptide levels of membrane CD47 receptor using mass spectrometry (MS, Thermo Orbitrap Velos Pro) at the University of Alabama at Birmingham (UAB) MS and proteomics core facility. It was found that the normal breast cells MCF-10A and 184B5 had no (0) or low (0.94) CD47 surface expression, respectively. The TNBC cells, including MDA-MB-468, MDA-MB-157, MDA-MB-453, MDA-MB-231, BT-20, and BT-549, had 2.04–5.29 of peptide counts of CD47 surface receptor (Figure 1B). Furthermore, to confirm the CD47 upregulation by chemotherapy, we treated MDA-MB-231 cells with 10 nM GC for 3 weeks. The Western blotting analysis showed that CD47 expression was increased in the GC-treated cells (Figure 1C, whole Western blot images see Supplementary Material). These data confirmed the literature finding that CD47 was upregulated by chemotherapies [29,30] and could be a suitable target for developing a targeting strategy (ADC) for TNBC.

### 3.2. Development and Characterization of Anti-CD47 mAb and ADC

**Figure 2:**
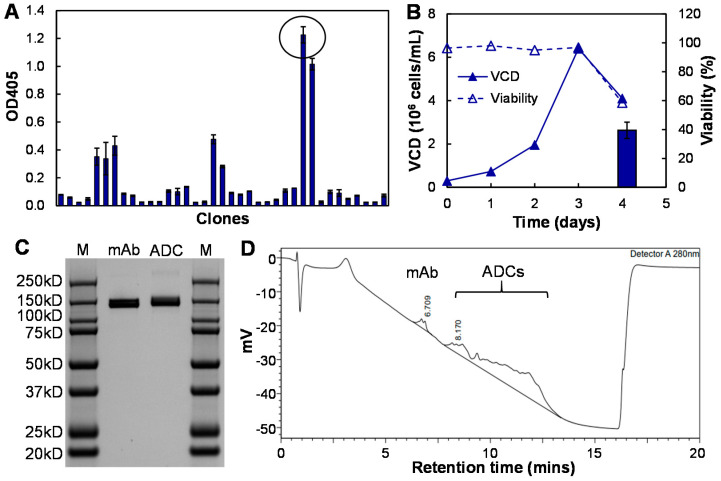
Anti-CD47 mAb and ADC development. (**A**) Rank of top anti-CD47 mAb clones based on the antibody titration using ELISA screening (data represent mean ± SEM, *n* = 3). (**B**) The anti-CD47 mAb production and hybridoma cell growth in shaker flask cell culture (data represent mean ± SEM, *n* = 3). Viable cell density (VCD): ▲, cell viability: Δ. (**C**) SDS-PAGE to confirm the integrity and purity of mAb and ADC. (**D**) Analysis of ADC synthesis efficiency (i.e., antibody–drug conjugation rate) using HPLC equipped with a MAbPac HIC-Butyl column.

The top CD47 hybridoma clone was screened with ELISA analysis using HEK293 expressed peptide (Gln19-Glu141) as the coating antigen (Figure 2A). We further adapted a CD47 mAb-producing hybridoma from an adherent culture to a suspension culture in serum free medium. The fed-batch bioproduction in Hybridoma-SFM reached maximal VCD of 6.4 × 10^6^ cells/mL and an average final titer of 32.5 mg/L (Figure 2B). The isotype analysis revealed that the developed CD47 mAb was IgG2b/kappa. Additionally, the affinity assay showed that the purified mAb had an equilibrium dissociation constant (K_D_) of 2.3 nM, suggesting a high surface receptor binding affinity of our anti-CD47 mAb.

The purified CD47 mAb with protein A column was conjugated with DM1 to construct ADC for TNBC treatment. The SDS-PAGE analysis confirmed the mAb’s molecular weight was 150 kDa and ADC was >150 kDa due to drug conjugation (Figure 2C), which is consistent with our previously established ADC conjugation platform [39]. The conjugation rate and DAR analysis using HPLC with the MAbPac HIC-Butyl column showed that our mAb–drug conjugation rate was >95% and the average DAR of the anti-CD47 ADC was 4–5 (Figure 2D). These data demonstrated that the anti-CD47 mAb-DM1 conjugate was successfully constructed with high yield, high conjugation efficiency, and good integrity. The developed mAb and ADC were further evaluated in this study for TNBC targeting and treatment both in vitro and in vivo.

### 3.3. Surface Binding and Tumor Targeting

**Figure 3:**
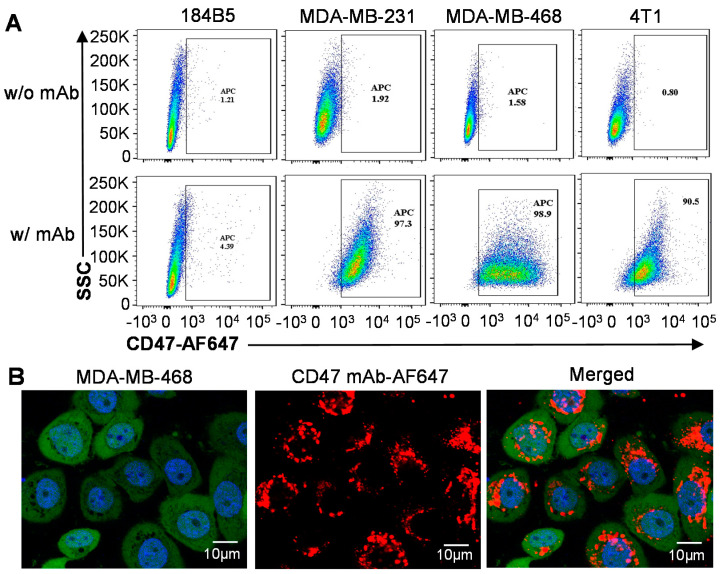
In vitro evaluation of surface binding and internalization of anti-CD47 mAb. (**A**) Flow cytometry to analyze the surface binding of anti-CD47 mAb to TNBC cells (MDA-MB-231, MDA-MB-468, and 4T1) and negative control cells (184B5). (**B**) Live-cell confocal microscopy imaging of the internalization of anti-CD47 mAb in MDA-MB-468 cells. Cytoplasm labeled with GFP (displayed as green), nucleus labelled with NucBlue (blue), and mAb-labeled with AF647 (red). Scale bar equals 10 µm.

Flow cytometry analysis: To assess the in vitro TNBC targeting capability of our anti-CD47 mAb, we first ran a flow cytometry analysis using normal breast 184B5 cells, human TNBC MDA-MB-231 and MDA-MB-468 cells, and mouse TNBC 4T1 cells (Figure 3A). The CD47 mAb showed high surface binding to MDA-MB-231 and -468 cells (97.3% and 98.9%, respectively) and 4T1 cells (90.5%) but low binding to normal 184B5 cells (4.39%), indicating a high TNBC targeting capability. Human CD47 (UniProtKB Q61735) and mouse CD47 (UniProtKB Q08722) have the same topology including three extracellular, five helical transmembrane, and three cytoplasmic domains. Protein BLAST analysis showed that the first extracellular domains of human and mouse CD47 receptors have similarity of 76%. The flow cytometry data suggested that our anti-CD47 mAb targeted both human and mouse TNBC cells with a high binding rate, so it is feasible to use a human TNBC xenograft mouse model to evaluate the tumor targeting of the new mAb in vivo.

Confocal microscopy: Furthermore, the live-cell confocal microscopy imaging showed that the AF647 labelled anti-CD47 mAb (displayed as red) effectively internalized into MDA-MB-468 cells via endocytosis and localized in the cytoplasm (detected with BacMam GFP) after mixing (Figure 3B). These data indicated that the CD47 mAb can effectively target and internalize into TNBC cells.

**Figure 4:**
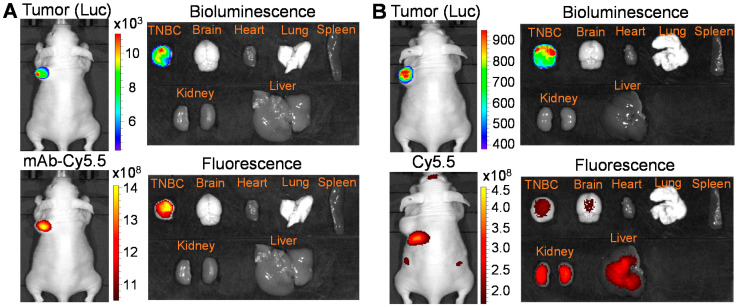
In vivo evaluation of TNBC targeting by anti-CD47 mAb in xenograft mice. (**A**) Live-animal and ex vivo IVIS imaging to confirm TNBC-specific targeting of CD47 mAb-Cy5.5. The IVIS images were collected at 24 h post tail vein injection of fluorescent dye Cy5.5-labelled mAb. (**B**) IVIS imaging showing the non-specific distribution of Cy5.5 dye in multiple organs such as kidney, liver, and brain in addition to the tumor.

IVIS imaging: The in vivo TNBC targeting of Cy5.5-labeled anti-CD47 mAb was evaluated using a MDA-MB-231-FLuc xenograft model. Live-animal IVIS imaging revealed that our mAb (indicated by Cy5.5 fluorescence) targeted and accumulated in the TNBC tumor (indicated by FLuc bioluminescence) within 24 h post i.v. injection (Figure 4A). There was no obvious distribution of mAb in the major organs such as brain, heart, lung, spleen, kidney, and liver. The same amount of Cy5.5 dye was used as control and i.v. injected into MDA-MB-231-FLuc xenograft model. The fluorescent dye without targeted mAb distributed in multiple locations such as brain, liver, and kidney. Altogether, these data suggested that the developed anti-CD47 mAb can effectively target TNBC cells or tumors, indicating its drug delivery capability.

### 3.4. In Vitro Anti-TNBC Cytotoxicity

**Figure 5:**
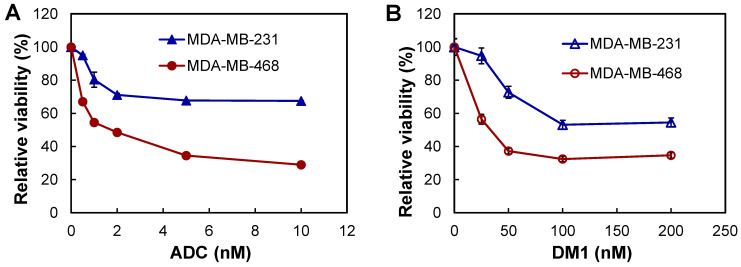
In vitro anti-TNBC cytotoxicity analysis of anti-CD47 ADC (**A**) and free drug DM1 (**B**). ●: MDA-MB-468, and ▲: MDA-MB-231. Data represent mean ± SEM, *n* = 3.

The in vitro anti-cancer cytotoxicity of the constructed anti-CD47 mAb-DM1 (ADC) was tested using TNBC MDA-MB-231 and MDA-MB-468 cell lines and free drug DM1 as the control. Multiple doses of ADC were tested including 0, 0.5, 1, 2, 5, and 10 nM. At the end of the 3-day assay, MDA-MB-468 cell viability decreased to 100%, 67%, 54%, 48%, 34%, and 29% and MDA-MB-231 cell viability decreased to 100%, 95%, 80%, 71%, 67%, and 67%, respectively (Figure 5A). The free drug at doses of 0, 25, 50, 100, and 200 nM reduced MDA-MB-468 cell viability to 100%, 56%, 37%, 32%, and 34% and MDA-MB-231 cell viability to 100%, 94%, 72%, 53%, and 54%, respectively (Figure 5B). The IC_50_ values of ADC were 0.3 nM and 0.8 nM and IC_50_ values of DM1 were 21 nM and 47 nM for MDA-MB-468 and MDA-MB-231, respectively. These data indicated that the conjugation of DM1 to anti-CD47 mAb increased the cytotoxicity, likely due to the high TNBC-targeting and drug delivery efficiency of mAb. It is also found that the MDA-MB-468 cell line was more sensitive to ADC and DM1 than was the MDA-MB-231.

### 3.5. In Vivo Anti-Tumor Efficacy

**Figure 6:**
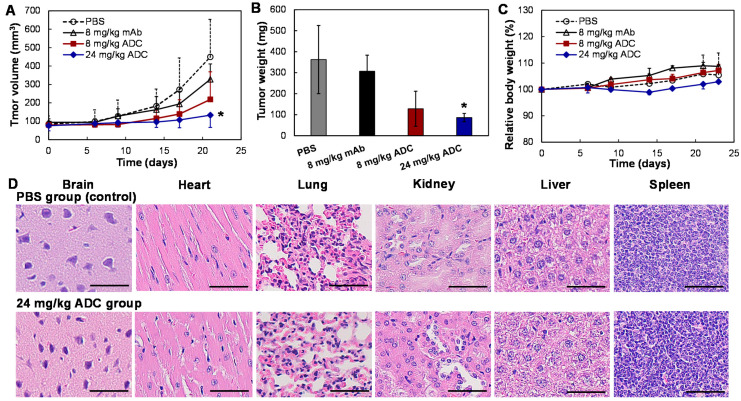
In vivo anti-tumor efficacy study of ADC in the TNBC (MDA-MB-231-FLuc) xenografted NSG mouse models. (**A**) Tumor volume changes with ADC treatment (data represent mean ± SEM, *n* = 5). PBS, mAb, or ADC were i.v. administrated into xenograft models on Days 6, 10, 14, and 18. Tumor size was measured with a caliper. Tumor volumes between the four groups were analyzed with mixed design ANOVA and multiple comparison. * *p* < 0.005. (**B**) Wet weight of the tumors excised from euthanized mice. * *p* ≤ 0.005. (**C**) Body weight changes. ○: PBS, ∆: 8 mg/kg mAb, ■: 8 mg/kg ADC, and ♦: 24 mg/kg ADC. (**D**) H&E staining of main organs, including brain, heart, lung, kidney, liver, and spleen. Scale bar equals to 50 µm.

To mimic chemotherapy in clinics, the xenograft mice were first treated with chemotherapy, i.e., 16 mg/kg of GC. When MDA-MB-231-FLuc xenografts reached ~100 mm^3^, mice were treated with anti-CD47 ADC (8 and 24 mg/kg), anti-CD47 mAb (8 mg/kg, control), or PBS (vehicle, control) on a Q4Dx4 schedule (i.e., Days 6, 10, 14, and 18) in four groups (*n* = 5). Figure 6A shows that the TNBC tumor growth rate was significantly reduced by 52% in the 8 mg/kg ADC group and 71% in the 24 mg/kg ADC group as compared to that of the PBS control group (*p* ≤ 0.005). The tumor weight in the 8 mg/kg mAb group was 307 ± 76 g, which showed no significant difference compared to that in PBS group (362 ± 162 g). The wet weight of the terminal tumor in the 8 and 24 mg/kg ADC groups was 65% and 77% lower than that of the PBS group (Figure 6B), which confirmed the treatment efficacy of ADC. As expected, there was no obvious difference among the four groups in overall body weight change (Figure 6C), suggesting that the toxicity of ADC was limited or well tolerated. To further evaluate the toxicity of TNBC treatment using anti-CD47 ADC, the important organs, such as brain, heart, lung, kidney, liver, and spleen, were collected when the mice were sacrificed, sectioned, and analyzed using hematoxylin and eosin (H&E) staining. As shown in Figure 6B, none of these organs in the mice treated with 24 mg/kg of ADC had obvious morphology changes or necrosis as compared to those of the PBS control group, indicating no evident off-target effects in vivo. These in vivo anti-TNBC efficacy data support the hypothesis that our anti-CD47 ADC is an effective drug delivery vehicle with minimal toxicity. Moreover, the anti-CD47 ADC was effective for chemotherapy-treated TNBC, which could be further developed as a novel therapy for TNBC.

### 3.6. General and Intratumoral Immune Response

**Figure 7:**
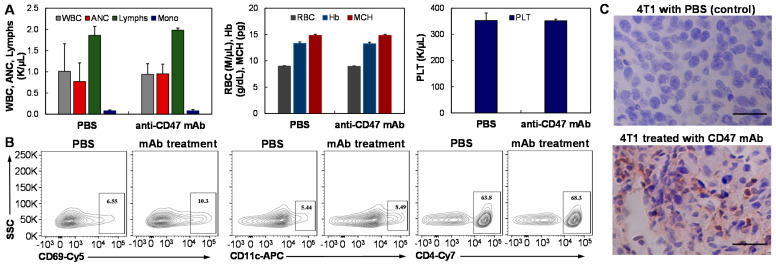
Immune response analysis of BALB/cJ mice post i.v. injection of anti-CD47 mAb. (**A**) Whole blood analysis showing minimal effect of anti-CD47 mAb on peripheral immunity. (**B**) Flow cytometry analysis of immune cells in lymph nodes indicating the increased level of CD69^+^ NK, CD11c^+^ DC, and CD4^+^ T cells. (**C**) IHC staining of the 4T1 xenograft tumor demonstrating obvious macrophage infiltration and phagocytosis in the tumor microenvironment.

We also tested the general immune response and immunity in the tumor microenvironment to anti-CD47 mAb in the 4T1 xenograft BALB/cJ model (*n* = 4) using whole blood analysis and flow cytometry analysis of lymph nodes and IHC staining of the tumor section, respectively. The mice carrying 50 mm^3^ xenograft were treated with 8 mg/kg anti-CD47 mAb. After two weeks, blood was collected through cardiac puncture. The whole blood analysis using HemaVet 950FS showed that the leukocytes (white blood cell, neutrophil, lymphocyte, monocyte, eosinophils), erythrocytes (red blood cell, hemoglobin, mean corpuscular hemoglobin), and thrombocytes (platelet) counts had no obvious difference between the mAb group and PBS control group (Figure 7A). These data indicated that our anti-CD47 mAb did not cause general peripheral immune toxicity. The lymph nodes were also harvested and dissociated for flow cytometry analysis of fresh cells. It was found that the CD69^+^ NK, CD11c^+^ DC, and CD4^+^ T cells were increased to 10.3%, 8.49%, and 68.3% in the mAb group from 6.55%, 5.44%, and 63.8% in the PBS control group (Figure 7B). Two weeks post mAb injection, the 4T1 xenograft tumors were collected, sectioned, and stained with anti-mouse CD11b antibody. The IHC staining images showed obvious macrophage infiltration into the 4T1 tumor and reduction of tumor cell density in the mAb treated group as compared to that of the PBS control group (Figure 7C), indicating the macrophage activation by blocking the “don’t eat me” signaling via SIRPα. These data demonstrated that our anti-CD47 mAb can cause an immune response in the tumor microenvironment but with low immune toxicity. In future, a full investigation of the immune function in the tumor microenvironment is needed in order to delineate the possible anti-TNBC immunity of this mAb.

## 4. Conclusions

In this study, we developed a new mAb and ADC that targets the extracellular domain of the surface receptor CD47 to treat TNBC post standard chemotherapy. The constructed anti-CD47 ADC effectively targeted TNBC cells and tumors, released potent drugs intracellularly, and significantly inhibited the tumor growth post GC treatment in a xenograft model with minimal side effects.

Drug resistance and distant metastasis pose major challenges in the standard chemotherapy of TNBC. Our developed anti-CD47 ADC has great potential to be further developed as a new targeted therapy for TNBC, especially post primary and chemotherapeutic treatments at time of recurrence. Furthermore, our anti-CD47 ADC could integrate multiple possible anti-cancer mechanisms: (1) direct TNBC cell death caused by the potent drug DM1, which has been used to treat other cancers via blocking of microtubulin polymerization; (2) increase of phagocytosis of CD47-positive TNBC cells (which needs further evaluation); and (3) potentially overcome the challenge of drug resistance in chemotherapy. These advantages can be combined with other therapies to clear TNBC cells in vivo.

Despite the promising results, there are some limitations in this study that warrant further investigation in the future. For instance, a further analysis of tumoral immunity of anti-CD47 ADC in immunocompetent models is needed to analyze the possible synergism of macrophage activation and ADC-delivered payload in TNBC treatment. The anti-tumor efficacy can be further improved by optimizing the dosage and treatment schedule. The pharmacokinetics and toxicity studies can provide therapy stability and systemic toxicity data. The patient-derived xenograft (PDX) model should be used to evaluate the treatment efficacy to heterogeneous TNBC. These investigations could provide more guidance for future potential pre-clinical or clinical studies. Additionally, we will investigate the application of our anti-CD47 mAb for targeted drug delivery via liposomes or other nanoparticles [62,63,64] for TNBC treatment.

## Data Availability

All data for this paper can be found in the text.

## Supplementary Materials

The following are available online at https://www.mdpi.com/article/10.3390/vaccines9080882/s1, Figure S1: Original western blots data.
