# Supplementary figures and images for: Anti-CD47 Monoclonal Antibody–Drug Conjugate: A Targeted Therapy to Treat Triple-Negative Breast Cancers
Source: Vaccines (Basel). 2021 Aug 10;9(8):882. doi: 10.3390/vaccines9080882 (PMC8402537; doi:10.3390/vaccines9080882)

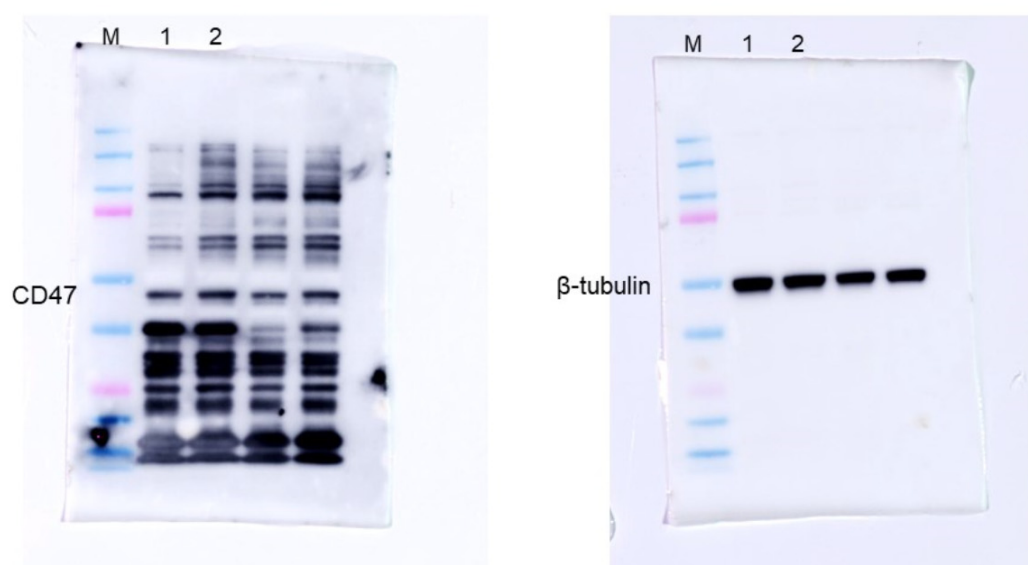

Figure S1. Original western blots data.

Supplement: Supplementary file 1 [file vaccines-09-00882-s001.zip › vaccines-1286452-supplementary.pdf]
